# Suicidal ideation in men during COVID-19: an examination of protective factors

**DOI:** 10.1186/s12888-023-04539-9

**Published:** 2023-01-18

**Authors:** Zac E. Seidler, Michael J. Wilson, John L. Oliffe, Krista Fisher, Rory O’Connor, Jane Pirkis, Simon M. Rice

**Affiliations:** 1 Orygen, Melbourne Parkville, Australia; 2 Centre for Youth Mental Health, The University of Melbourne, Parkville, Australia; 3 Movember, Melbourne, Australia; 4 School of Nursing, University of British Columbia, Vancouver, Canada; 5 Department of Nursing, The University of Melbourne, Parkville, Australia; 6 Suicidal Behaviour Research Laboratory, Institute of Health & Wellbeing, University of Glasgow, Glasgow, UK; 7 Centre for Mental Health, Melbourne School of Population and Global Health, The University of Melbourne, Parkville, Australia

**Keywords:** Suicide, Suicidal thoughts, Men, Masculinity, COVID-19, Resilience, Coping, Economic crisis, Protective factors, Interpersonal factors

## Abstract

**Background:**

Men account for three-quarters of all suicide deaths in many Western nations including Australia. Whilst extensive research has examined risk factors for suicidal ideation and behaviour in men, protective factors remain underexplored, particularly social support, resilience and coping behaviours. Such factors are important to examine particularly in the context of COVID-19, where enforced isolation (among other negative lifestyle effects) has created widespread risk for the development of suicidal ideation. This mixed-methods study aimed to examine associations of various protective factors with suicidal ideation in men, using data from an online survey conducted during the COVID-19 pandemic. In addition, we aimed to qualitatively investigate men’s self-reported protective strategies when experiencing suicidal thoughts and behaviour.

**Methods:**

A convenience sample of 700 men (age *M* = 50.3 years; *SD* = 15.2 years) responded to an online survey including quantitative measures of suicidal ideation, planning and attempt, alongside employment and relationship status, coping, social support, resilience, and a qualitative free-text item gauging men’s self-reported protective strategies. Multinomial logistic regression was applied to compare odds of sub-categories of suicide risk (ideation; planning) according to protective factors. Qualitative responses were analysed via thematic analysis.

**Results:**

Men in a relationship, and those lower in emotion-focused and avoidant coping reported lower odds of suicidal ideation. Maintaining employment throughout the pandemic was protective against suicidal ideation and planning; as was greater perceived social support from friends. Greater self-reported resilience was protective against suicidal ideation and planning. Qualitative analyses led to the development of two themes: *coping and connecting*, reflecting men’s intra- and interpersonal management strategies; and *sustaining selflessness*, where men’s imaginings of the collateral damage of their suicidal behaviour was protective against action on suicidal thoughts or plans.

**Conclusions:**

Findings of this study speak to the nuanced roles of interpersonal connections, resilience and coping behaviours in protecting against suicidal ideation and planning in men. In addition, qualitative insights further cement men’s identification with familial protector and/or provider roles as protective against suicidal action.

## Suicide in men

Male suicide remains a major public health concern throughout the world [1]. Consistent with data from other countries (e.g., [2]), men in Australia die by suicide at a rate approximately three times that of women [3]. It is therefore essential for research to focus on understanding the development of suicidal thoughts and behaviour in men, alongside risk and protective factors [4]. Such research is particularly warranted in the context of the novel coronavirus (COVID-19) pandemic, where enforced isolation, unemployment and interpersonal strain has led to an emerging and ongoing mental health burden [5, 6]. These are particularly concerning risk factors for suicide and may have unique implications for men [7]. Despite early evidence suggesting rates of suicide death have not increased over the course of the pandemic [8], evidence indicates increased rates of suicidal ideation relative to pre-pandemic indices [9]. Moreover, risk of suicide in the context of global crises tends to increase once the immediate crisis has passed [10, 11].

Suicide risk is understood to exist on a continuum from suicidal thoughts (i.e., thoughts of ending one’s life) to suicidal action (i.e., acting out attempts to take one’s life; [12]). Prominent theory-based scholarship offers useful frameworks for understanding *how* and *why* individuals, irrespective of gender, progress through this continuum. O’Connor’s [13, 14] Integrated Motivational-Volitional (IMV) model of suicidal behaviour incorporates factors from existing models like the Interpersonal Theory of Suicide (IPTS; [15]) that moderate the progression through stages of ideation and subsequently to suicidal action. Specifically, IPTS constructs of thwarted belongingness (i.e., being entirely alone) and burdensomeness (i.e., the sense that people are “better off without me”) are categorised as ‘motivational moderators’ in the IMV model. These factors are seen to contribute to the formation of suicidal ideation and intent. The subsequent transition from this intent to suicidal behaviour is moderated by volitional factors such as capability, impulsivity, devising a plan and access to means [14], factors which are thought to readily explain transitions across the suicide continuum in men [16].

Whilst connections between the above constructs and the continuum of suicide risk have been validated in past research (e.g., [17]), there remains scope to better understand the progression from suicidal thoughts to suicidal behaviours in men, and the factors that contribute to, or protect against, the escalation of distress into suicidal thoughts or behaviours [13]. This is particularly important not only because of the disproportional burden of male suicide [2], but also because the progression from ideation to action is thought to occur more rapidly in men [18]. One aspect of suicide risk that requires better delineation in future research is suicide planning. Modern perspectives on the development of suicidal behaviour argue planning for suicide is not a prerequisite for the progression from ideation to suicide attempt [19], where other scholars highlight that planning is indicative of “active” as opposed to “passive” ideation [20]. Arguably the IMV model is the only theory to propose planning as a moderator, as opposed to a distinct stage of risk between ideation and attempt [13]. Suicide planning is nevertheless a clear risk factor for subsequent suicidal action among those with ideation [21, 22]. Indeed, Nock and colleagues [23] found cross-national evidence that the conditional probability of suicide attempt among those with a plan is 56.0%, relative to 15.4% among those without a plan. Given many male suicides are thought to occur impulsively, incorporating assessment of suicide planning into studies of men’s risk of suicidal thoughts in relation to risk and protective factors is an important avenue to better identify groups of men who are at elevated risk of suicidal behaviour.

Several qualitative studies with male participants have identified a period of heightened risk of suicidal action, where ideation has progressed to the point of clear planning for suicide in the lead up to suicidal action [24–26]. Thus, it is important to better identify and intervene with those men whose suicidal ideation incorporates planning, with growing evidence suggesting active ideation and planning can be prerequisites to a suicide attempt [15, 27, 28]. Prior research has found that suicide-specific cognitions successfully delineate individuals with suicidal ideation alone, relative to those with ideation and planning [29]. Yet to date, studies that aim to examine risk and protective factors for suicide in men have not captured suicide planning as an outcome. Clearer assessment of planning in relation to constructs that might delineate planning from ideation alone is needed to uncover potential intervention pathways, given these constructs hav*e* been conflated or entirely absent in previous reviews [30]. Of note, to our knowledge the only prior study to comprehensively examine risk and protective factors for suicide in men did not assess suicide planning, focusing instead on ideation and attempt as outcomes [31].

## Factors that are protective against suicide in men

Greater consideration is needed of the different protective psychological and/or situational factors associated with male suicidal thoughts and behaviours, particularly in the context of the pandemic which elicited widespread employment and social disruption [7, 32]. Employment is known to be protective against suicide in men: population-level data have indicated risk of suicide among unemployed men, alongside an increased risk of self-harm among men not in the labour force [33]. Men’s socialisation to be primary familial providers and the common occurrence for men to derive self-worth from their employment [34], highlights the need to appraise unemployment in relation to the continuum of suicide risk in men. Being married or partnered is also known to protect men against suicide [35], and relationship strain has been widely reported throughout the pandemic [36, 37]. The risk of men’s suicide amid separation also increases when experienced in combination with mental ill-health and substance use [38], where socialized dominant masculine ideals of self-reliance can stymie the seeking of social support in the context of intimate partner separation [39]. Therefore, there is scope to achieve greater nuance in our understanding of the links between maintaining employment, relationship status and components of suicide risk in men. The vast majority of research on these subjects has retrospectively compared men in and out of relationships, and employed or unemployed men according to risk of suicide death [33, 35], however understanding the links between these factors and different points along the suicide continuum remains limited.

Emerging evidence also suggests social support as an important protective factor against suicide among men. Higher levels of social support are associated with reduced likelihood of men reporting a suicide attempt, compared to suicidal ideation alone [31], and a sense of social obligation to others (i.e., friends or family) is known to be protective against suicide attempts in men [40]. However, to our knowledge no research among men has compared associations between various forms of social support (i.e., from partners, family or friends) and categories of suicide risk (i.e., ideation and/or planning). A prior study of risk and protective factors for suicide ideation relative to attempt in men only assessed social support in general, without specific delineation by sources of social support [31]. It is important to capture this nuance within the context of the pandemic, where opportunities for normative avenues of social support have been restricted, with associated mental health consequences [6, 41]. Additionally, connections between masculine socialisation and men’s diverse practices of social engagement result in reliance on different sources for varied types of support: friends are often relied on for ‘instrumental’ support and shared activities, whereas romantic partners are often relied upon as a primary source of emotional support [39].

Similarly, higher levels of resilience (i.e., the capacity to adapt to adversity and stress) have been associated with reduced risk of suicide attempts among men [42]. Research amongst male veterans has also found that higher resilience at baseline significantly predicts lower suicidal thoughtsat three-year follow-up [43]. Along with resilience, certain coping strategies may also interrupt the escalation of suicide riskin men. Commonly, approach-oriented activities that are either problem-focused (e.g., planning, positive reframing) or emotion focused (e.g., seeking emotional support, acceptance) are known to reduce risk of suicidal ideation [44], where avoidance-oriented strategies (e.g., withdrawing, increased alcohol and/or drug use) appear to increase risk of suicide [45]. Past qualitative work has also explored the idea that many men cope through connectedness or via a sense of altruistic responsibility for the welfare of others, with suicidal men refraining from acting on suicide plans due to their sense of obligation toward their loved ones [24, 46, 47]. Social support, resilience and coping therefore warrant investigation in the context of COVID-19, where a suite of adverse experiences (e.g., relationship stress; job loss) that confer greater risk for suicide are known to have affected men [37]. This level of depth of concurrent examination of risk and protective factors for male suicide has not been achieved to-date in the available literature [31]. Accompanying this examination with a qualitative analysis of protective factors that keep men safe from suicide is also needed, and has not been conducted to-date in prior research on male suicide risk. This will help to build upon quantitative examination of associations of social support, coping, and resilience with risk of suicide. Allowing men the opportunity to describe the elements keeping them safe lends itself to a nuanced examination of factors that prohibit in-the-moment suicidal action, beyond those protective factors that mitigate suicidal distress more broadly.

## Current study

This exploratory mixed methods study aimed to examine, in the context of COVID-19, associations between risk factors (i.e., job loss; relationship breakdown) and protective factors (social support; coping; resilience) and suicidal ideation and/or planning in a sample of Australian men. Qualitative data regarding men’s protective factors when experiencing suicidal thoughts or behaviour were also gathered, allowing complementary understanding of the factors that kept them safe throughout the pandemic.

## Methods

### Participants and procedure

Between 25th October and 29 December 2021, Australian self-identifying men aged 16 + were invited to take part in an online survey focused on their mental health experiences during the pandemic. A link to the Qualtrics survey was shared using paid Facebook advertisements. Individuals who accessed the link were initially presented with a plain language statement and consent form. Consent was collected via a yes/no survey item, and following this, the survey contained a mix of Likert scale-type questions and free-text qualitative questions capturing demographics, suicidal thoughts and behaviour and mental health measures, COVID-19-related stressors, help-seeking behaviours and protective factors. Participants who completed the survey were invited to enter a draw to win a $500 gift voucher. Ethics approval for this study was granted by the University of Melbourne Faculty of Medicine, Dentistry and Health Sciences Human Research Ethics Committee (ID: 1,956,099.3).

### Measures

Sample demographics were measured via a series of self-report items assessing age, gender identity, sexual orientation, place of residence (i.e., metropolitan, regional or rural/remote), education level. Measures of suicidal thoughts and behaviour, and risk and protective factors are described below, as is the question that elicited free text responses.

#### Suicidal thoughts and behaviour

Suicidal thoughts and behaviour were assessed using three binary-response items sourced from the *Ten to Men* study, Australia’s national longitudinal study exploring the social and emotional wellbeing of boys and men [48]. Ideation was assessed with, “*Since March 2020 (the beginning of the pandemic), have you seriously thought about killing yourself?*” Planning (or ‘active ideation’) was assessed with, “*Since March 2020 (the beginning of the pandemic), have you made a plan about how you would kill yourself?*” Finally, any attempts at suicide throughout COVID-19 was assessed with, “*Since March 2020 (the beginning of the pandemic), have you tried to kill yourself?*” All items included two response options (*yes, no*)*.*

#### Protective factors

##### COVID-19 era employment status

To assess employment status during COVID-19, participants were asked, “*Have you lost your job due to the COVID-19 pandemic?*” Response options were as follows: *no, but my hours were reduced* (1); *no, but job loss is expected* (2); *no, but I’ve had to work from home* (3); *yes, and I haven’t found another job* (4); *yes, and I have found another job* (5); *no my work has not been affected* (6); *I don’t work at all (not in the labour force/retired)* (7). For this study, due to low counts across the original sub-categories, the variable was collapsed into a categorical variable with three levels: *job loss experienced* coded (1); *not in the labor force/retired* coded (2); and *no job loss experienced* coded (3). This item was adapted from Ogrodniczuk and colleagues [37].

##### Relationship status

To assess relationship status, participants were asked, “*What is your current relationship status?*” Response options were as follows: *Single/never married* (1); *partnered* (2); *married/de-facto* (3); *single—separated/divorced* (4); *widowed* (5); and *other* (6). For this study, the original variable was collapsed to the following three categories: *Single/never married* (1); *Separated/divorced/widowed* (2); and *married/partnered/de-facto* (3).

##### Coping

Coping was assessed using the Brief Coping Orientation to Problems Experienced inventory (Brief-COPE; [49]). The Brief-COPE consists of three subscales that assess different coping styles: emotion-focused coping (e.g., “*I’ve been saying things to let my unpleasant feeling escape*”); problem-focused coping (e.g., “*I’ve been taking action to try and make the situation better*”); and avoidant coping (e.g., “*I’ve been using alcohol or other drugs to help me get through it*”). Participants rate their recent coping behaviours according to 28 items on a scale of 1 (*I haven’t been doing this at all*) to 4 (*I’ve been doing this a lot*). Responses are summed, with higher subscale scores indicating greater levels of the respective coping styles. Internal consistencies of each subscale varied in this study (emotion-focused coping α = 0.67; problem-focused coping α = 0.85; avoidant coping α = 0.67); notwithstanding this, the reliability and validity of the scale in assessing coping irrespective of context and stressor has been previously demonstrated [50].

##### Social support

Social support was assessed using the Multidimensional Scale of Perceived Social Support (MSPSS; [51]). The scale contains 12 items (e.g., “*I have friends with whom I can share my joys and sorrows*”), each rated on a scale of 1 (*very strongly disagree*) to 7 (*very strongly agree*), which measure perceived social support from a significant other (i.e., partner), family, and friends. Responses are summed, with higher scores indicating greater perceived social support. The internal consistency and construct validity of the scale have been established previously [51]. Each of the *significant other* (α = 0.96), *family* (α = 0.94) and *friends* (α = 0.95) subscales were used in this study, all with excellent internal consistencies.

##### Resilience

Resilience was assessed using the Brief Resilience Scale (BRS; [52]). The scale consists of six items (e.g., “*I tend to bounce back quickly after hard times*”), which are rated on a scale of 1 (*strongly disagree*) to 5 (*strongly agree*). Higher average scores indicate greater resilience. The internal consistency and convergent validity of the BRS has been demonstrated previously [52, 53]. Internal consistency was good in this study (α = 0.87).

##### Free text question

Participants who endorsed any of the items assessing suicidal thoughts or behaviour based on the items above, were asked to respond in free text to the following question: *What are the key protective factors (things that have kept you safe) when experiencing suicidal thoughts/behaviour?*

### Quantitative data analysis

Data analysis was conducted using SPSS Version 27. Firstly, based on responses to the three suicidal thoughts/behaviour items, a variable was created and examined to identify proportions of participants across four groups: no suicidal thoughts or behaviour (i.e., *no* to all items; coded 1); suicidal ideation only (i.e., *yes* to ideation, *no* to planning and attempt; coded 2); suicidal ideation and planning (i.e., *yes* to ideation and planning, *no* to attempt; coded 3) and suicidal ideation, planning and attempt (i.e., *yes* to ideation, planning and attempt; coded 4). Frequencies across category membership were examined.

Next, separate univariate multinomial logistic regression analyses were run for each variable, with suicide sub-category membership as the outcome variable. These analyses yielded unadjusted odds ratios and corresponding significance values for each covariate. Finally, all measures were included in a multinomial logistic regression model to obtain fully-adjusted odds ratios for each variable. Odds of ideation relative to no suicidal thoughts or behaviours were examined followed by ideation and planning relative to no suicidal thoughts or behaviours; finally, the reference category was set to ideation to obtain odds of suicide planning relative to ideation alone. For all quantitative analyses, as this was an exploratory study, *p* < 0.05 was adopted as the threshold for statistical significance. Listwise deletion was applied to remove missing data.

### Qualitative data analysis

Free text responses to the question regarding protective factors were analysed using inductive thematic analysis, across stages of coding and theme development [54], following recommendations for the conduct of qualitative analysis using survey data [55]. All responses were initially read and re-read in depth by one author (MW) to obtain a sense of familiarity with the data. Responses were subsequently downloaded to a spreadsheet, and missing responses removed to facilitate analysis. Independent coding was first conducted by MW, where descriptive codes representing distinct units of meaning were developed and grouped to encompass similar responses. These initial codes were then refined under higher-order categories in consultation with a second author (ZS), where any disagreements regarding theme membership of various data and codes was discussed to reach consensus. For example, initially all codes reflecting the role of other people as protective were grouped under a single theme labelled *Interpersonal connections*, however, it became clear that further distinction was necessary regarding the nature of friends versus family in their respective protective roles. The finalised thematic structure was then reviewed in consultation with authors JLO and SR, and theme names and exemplar quotes were decided in consultation throughout the manuscript writing and revision process. All participants who were included in the quantitative component and provided a valid response to the free-text protective factors item, were included in qualitative analysis. Responses were included if they conveyed meaning irrespective of length, as even responses of very few words contributed to our understanding of men’s self-reported protective factors when experiencing suicidal thoughts or behaviours.

## Results

In total, 812 participants responded to the survey. A sub-sample of 700 participants responded to the items assessing suicidal thoughts or behaviours throughout the COVID-19 pandemic and were thus included in the current sample. Those who responded to the relevant items to this study did not differ from non-respondents in terms of age (*p* = 0.975); sexuality (*p* = 0.982); gender identity (*p* = 0.814); education level (*p* = 0.052) or place of residence (*p* = 0.122). The proportions of participants across categories of suicidal ideation, planning and attempt are reported in Table 1 below.

**Table 1:** Proportions of participants across categories of suicide risk

Category	*n*	%
No suicidal thoughts or behaviours	497	61.2
Ideation only	87	10.7
Ideation and planning	104	12.8
Ideation, planning and attempt^a^	12	1.5

^a^For the current analyses *n* = 12 who reported attempting suicide were combined with the ideation and planning group

For all subsequent analysis, given low sample sizes in sub-categories, only those reporting no suicidal thoughts or behaviours, ideation only and ideation and planning were included. Those reporting ideation, planning and attempt (*n* = 12) were included in the ideation and planning category for the purposes of further analysis focusing on delineating no suicidal thoughts or behaviours (*n* = 497), ideation alone (*n* = 87), and ideation and planning (*n* = 116). Descriptive statistics for all measures are included in Table 2 below.

**Table 2:** Frequencies, means and standard deviations of all variables across suicide risk category membership

	**Full sample** *N* = 700	**No suicidal thoughts or behaviours** *n* = 497	**Ideation only** *n* = 87	**Ideation + planning**^**1**^ *n* = 116
**Demographics**				
Age *M* (*SD*)	50.3 (15.2)	50.9 (15.0)	48.1 (15.8)	49.5 (15.5)
Sexual identity *n* (%)^2^				
Heterosexual	495 (70.7)	354 (71.2; 71.5)	58 (66.7; 11.7)	83 (71.6; 16.8)
Gay	150 (21.4)	107 (21.5; 71.3)	18 (20.7; 12.0)	25 (21.6; 16.7)
Bisexual	43 (6.1)	33 (6.6; 76.7)	6 (6.9; 14.0)	4 (3.4; 9.3)
Other	12 (1.7)	3 (0.6; 25.0)	5 (5.7; 41.7)	4 (3.4; 33.3)
Gender identity *n* (%)^2^				
Cisgender man	692 (98.9)	494 (99.4; 71.4)	84 (96.6; 12.1)	114 (98.3; 16.5)
Transgender man	8 (1.1)	3 (0.6; 37.5)	3 (3.4; 37.5)	2 (1.7; 25.0)
Education level *n* (%)^2^				
University educated	400 (57.1)	288 (57.9; 72.0)	51 (58.6; 12.8)	61 (52.6; 15.3)
Non-university educated	300 (42.9)	209 (42.1; 69.7)	36 (41.4; 12.0)	55 (47.4; 18.3)
Place of residence *n* (%)^2^				
Metropolitan	459 (65.6)	345 (69.4; 75.2)	50 (57.5; 10.9)	64 (55.2; 13.9)
Non-metropolitan	241 (34.4)	152 (30.6; 63.1)	37 (42.5; 15.4)	52 (44.8; 21.6)
**Protective factors**				
Employment status *n* (%)*^2^				
No job loss experienced	388 (64.7)	296 (70.5; 76.3)	47 (60.3; 12.1)	45 (44.1; 11.6)
Not in labor force/retired	134 (22.3)	82 (19.5; 61.2)	19 (24.4; 14.2)	33 (32.4; 24.6)
Job loss experienced	78 (13.0)	42 (10.0; 53.8)	12 (15.4; 15.4)	24 (23.5; 30.8)
Relationship status *n* (%)*^2^				
Single (never married)	147 (21.0)	89 (17.9; 60.5)	25 (28.7; 17.0)	33 (28.4; 22.4)
Separated/divorced/widowed	117 (16.7)	67 (13.5; 57.3)	23 (26.4; 19.7)	27 (23.3; 23.1)
Married/partnered/de-facto	436 (62.3)	341 (68.6; 78.2)	39 (44.8; 8.9)	56 (48.3; 12.8)
Significant other social support	4.87 (1.97)	5.19 (1.84)	4.15 (2.08)	4.05 (2.03)
Family social support	4.19 (1.85)	4.53 (1.75)	3.47 (1.90)	3.32 (1.78)
Friends social support	4.29 (1.77)	4.59 (1.65)	3.89 (1.80)	3.38 (1.84)
Problem-focused coping	18.50 (5.38)	18.33 (5.41)	18.77 (4.86)	19.00 (5.64)
Emotion-focused coping	26.11 (5.51)	25.51 (5.50)	27.66 (4.90)	27.33 (5.58)
Avoidant coping	15.31 (4.23)	14.23 (3.61)	17.46 (3.98)	18.06 (4.87)
Resilience	3.21 (0.97)	3.45 (0.90)	2.71 (0.84)	2.62 (0.93)

^*^ = Columns do not sum to full sample *N* due to missing data. 1 = this category includes *n* = 12 individuals who reported suicidal ideation, planning and attempt. 2 = column percentage; row percentage

### Multinomial logistic regression model

The overall model fit was good: χ^2^(1162) = 1077.01, *p* = 0.964, Nagelkerke R^2^ = 0.383. Furthermore, a test of the full model against an intercept-only model was significant: χ^2^(36) = 221.235, *p* < 0.001, indicating the set of covariates effectively distinguished participants across suicide risk category membership. Unadjusted (i.e., univariate) and fully-adjusted (multivariate) odds ratios and 95% CI’s for paired comparisons between suicide risk categories are reported in Table 3 and described in turn below.

**Table 3:** Multinomial logistic regression of suicide category membership by demographic and protective factors

	**Ideation vs no suicidal thoughts or behaviours**				**Ideation + planning**^**1**^** vs no suicidal thoughts or behaviours**				**Ideation + planning**^**1**^** vs ideation**			
**Demographics**	Unadjusted OR [95% CI]	*p*	Fully adjusted OR [95% CI]	*p*	Unadjusted OR [95% CI]	*p*	Fully adjusted OR [95% CI]	*p*	Unadjusted OR [95% CI]	*p*	Fully adjusted OR [95% CI]	*p*
Age	0.99 [0.97, 1.00]	.109	0.99 [0.97, 1.02]	.609	0.99 [0.98, 1.01]	.359	0.98 [0.96, 1.00]	.070	1.01 [0.99, 1.02]	.519	0.99 [0.96, 1.01]	.273
Sexuality (reference: heterosexual)												
Gay	1.03 [0.58, 1.82]	.928	0.79 [0.40, 1.54]	.485	1.00 [0.61, 1.64]	.989	0.69 [0.36, 1.29]	.243	0.97 [0.49, 1.94]	.933	0.87 [0.40, 1.89]	.726
Bisexual	1.11 [0.45, 2.77]	.823	0.80 [0.26, 2.47]	.704	0.52 [0.18, 1.50]	.225	0.32 [0.08, 1.27]	.105	0.47 [0.13, 1.73]	.253	0.40 [0.09, 1.83]	.236
Other	10.17 [2.37, 43.72]	.002	4.72 [0.73, 30.66]	.104	5.69 [1.25, 25.90]	.025	3.34 [0.44, 25.31]	.244	0.56 [0.14, 2.17]	.401	0.71 [0.12, 4.06]	.697
Gender identity (reference: transgender man)												
Cisgender man	0.17 [0.03, 0.86]	.032	0.14 [0.02, 1.02]	.052	0.35 [0.06, 2.10]	.248	0.26 [0.03, 2.34]	.230	2.04 [0.33, 12.46]	.442	1.91 [0.27, 13.57]	.519
Education level (reference: not university educated)												
University educated	1.03 [0.65, 1.63]	.907	1.22 [0.68, 2.17]	.506	0.81 [0.54, 1.21]	.294	1.23 [0.71, 2.13]	.452	0.78 [0.45, 1.37]	.393	1.01 [0.52, 1.97]	.967
Place of residence (reference: non-metropolitan)												
Metropolitan	0.60 [0.37, 0.95]	.029	**0.53 [0.30, 0.96]**	**.037**	0.54 [0.36, 0.82]	.004	**0.50 [0.29, 0.87]**	**.015**	0.91 [0.52, 1.60]	.744	0.93 [0.49, 1.80]	.838
**Protective factors**												
Employment status (reference: job loss experienced)												
No job loss experienced	0.56 [0.27, 1.13]	.106	0.85 [0.38, 1.87]	.682	0.27 [0.15, 0.48]	< .001	**0.38 [0.19, 0.77]**	**.007**	0.48 [0.21, 1.07]	.073	0.45 [0.19, 1.05]	.066
Not in labor force/retired	0.81 [0.36, 1.83]	.613	1.11 [0.42, 2.93]	.830	0.70 [0.37, 1.34]	.286	1.26 [0.55, 2.89]	.593	0.87 [0.36, 2.12]	.757	1.13 [0.41, 3.09]	.813
Relationship status (reference: married/partnered/de facto)												
Single (never married)	2.46 [1.41, 4.27]	.001	1.60 [0.70, 3.62]	.263	2.26 [1.38, 3.68]	.001	1.40 [0.63, 3.10]	.410	0.92 [0.46, 1.78]	.803	0.88 [0.34, 2.24]	.782
Separated/divorced/widowed	3.00 [1.68, 5.35]	< .001	**2.67 [1.21, 5.89]**	**.015**	2.45 [1.45, 4.16]	.001	1.82 [0.84, 3.95]	.130	0.82 [0.41, 1.63]	.567	0.68 [0.28, 1.67]	.405
SO social support	0.77 [0.68, 0.86]	< .001	0.93 [0.76, 1.14]	.505	0.75 [0.67, 0.83]	< .001	0.98 [0.81, 1.19]	.839	0.98 [0.85, 1.12]	.737	1.05 [0.84, 1.32]	.671
Family social support	0.73 [0.64, 0.83]	< .001	0.86 [0.71, 1.05]	.135	0.69 [0.62, 0.78]	< .001	0.88 [0.73, 1.06]	.190	0.96 [0.82, 1.12]	.569	1.02 [0.82, 1.28]	.842
Friends social support	0.79 [0.69, 0.91]	.001	0.94 [0.76, 1.17]	.599	0.67 [0.60, 0.76]	< .001	**0.76 [0.62, 0.93]**	**.007**	0.85 [0.72, 0.99]	.046	0.80 [0.63, 1.02]	.074
Problem-focused coping	1.02 [0.97, 1.06]	.499	1.01 [0.94, 1.08]	.753	1.02 [0.98, 1.06]	.250	**1.08 [1.01, 1.16]**	**.019**	1.01 [0.96, 1.06]	.770	1.07 [0.99, 1.16]	.087
Emotion-focused coping	1.08 [1.03, 1.12]	.001	**1.07 [1.00, 1.15]**	**.048**	1.06 [1.02, 1.11]	.002	1.02 [0.95, 1.09]	.662	0.99 [0.94, 1.04]	.681	0.95 [0.88, 1.02]	.165
Avoidant coping	1.22 [1.15, 1.30]	< .001	**1.12 [1.05, 1.21]**	**.002**	1.26 [1.19, 1.33]	< .001	**1.18 [1.10, 1.27]**	**< .001**	1.03 [0.97, 1.10]	.372	1.05 [0.97, 1.14]	.203
Resilience	0.40 [0.30, 0.54]	< .001	**0.52 [0.38, 0.72]**	**< .001**	0.36 [0.28, 0.47]	< .001	**0.48 [0.35, 0.66]**	**< .001**	0.90 [0.65, 1.24]	.514	0.91 [0.63, 1.31]	.617

**Bold** = *p* < .05. OR = odds ratio. SO = significant other. 1 = this category includes *n* = 12 individuals reporting suicide ideation, planning and attempt

#### Odds of suicidal ideation relative to no suicidal thoughts or behaviours

Lower odds of suicidal ideation were observed for metropolitan-residing relative to non-metropolitan participants (OR = 0.53 [0.30, 0.96], *p* = 0.037). Greater odds of ideation were also observed for separated, divorced or widowed men relative to married or partnered men (OR = 2.67 [1.21, 5.89], *p* = 0.015). Odds of suicidal ideation were greater alongside increases in both emotion-focused coping (OR = 1.07 [1.00, 1.15], *p* = 0.048) and avoidant coping (OR = 1.12 [1.05, 1.21], *p* = 0.002). Additionally, higher resilience protected against suicidal ideation (OR = 0.52 [0.38, 0.72], *p* < 0.001). Neither employment nor any forms of social support significantly delineated those who experienced suicidal ideation relative to no suicidal thoughts or behaviours.

#### Odds of suicidal ideation and planning relative to no suicidal thoughts or behaviours

As above, residing in a metropolitan area appeared to be protective against suicide planning relative to no suicidal thoughts or behaviours(OR = 0.50 [0.29, 0.87], *p* = 0.015), as did the maintenance of employment throughout the pandemic (OR = 0.38 [0.19, 0.77], *p* = 0.007). Higher levels of social support from friends was protective against suicide planning (OR = 0.76 [0.62, 0.93], *p* = 0.007). Conversely, greater odds of experiencing suicide planning were observed with higher levels of problem-focused coping (OR = 1.08 [1.01, 1.16], *p* = 0.019) and avoidant coping (OR = 1.18 [1.18, 1.27], *p* < 0.001). Higher resilience was protective against experiencing suicide planning (OR = 0.48 [0.35, 0.66], *p* < 0.001). Relationship status was unrelated to odds of experiencing suicide planning relative to no suicidal thoughts or behaviours.

#### Odds of suicidal ideation and planning relative to suicidal ideation

No covariates significantly delineated the likelihood of experiencing suicide planning relative to ideation alone (see Table 3).

### Qualitative results

Following the exclusion of two blank responses, 201 participants who experienced suicidal ideation since the outset of the pandemic provided free text responses about the key protective factors that kept them safe. Two broad themes were developed from the data and are discussed in detail below. In summary, the first theme, *Coping and Connecting,* encompassed the importance of building up internal coping resources (e.g. perspective taking; distress tolerance) alongside knowing when and how to connect with others as adaptive attempts to keep oneself safe and salve distress. The second theme, *Sustaining Selflessness,* spoke to a fear of the likely pain involved in a suicide attempt and the maladaptive behaviours used to cope with these thoughts (e.g., drug and alcohol use), alongside a constant foresight of the dire and long-lasting ramifications of their death on their loved ones.

#### Coping and connecting

This theme, encompassing responses from 169 participants, centered on the importance of participants’ internal (i.e., psychological) and external (i.e., social) coping resources as protective. These were often described as a work in progress, learned through trial and error over time and often shifting in effectiveness depending on the nature of the suicidal thoughts.

Firstly, common in this theme was mention of a sense of determination and resilience among participants to fight the metaphorical ‘battle’ against their distress. Challenging a framing among some men of suicide as a masculine act of taking back control [46], for many participants, succumbing to the weight of suicidal thoughts was negatively framed as *“cowardice”,* failure and an impulsive loss of control. One participant reported “*I hate being defeated*”, and another refused to “*let the bastards win*” thus reframing a potential deficit as a call to action with steely resolve. Another participant provided a striking description of his inner determination, noting how the act of externalising his distress offered him a tangible conquest worth fighting:

*“When it gets bad I externalise my depression and anxiety. I treat it like a wild animal that is threatening those I love. No one gets hurt because of me so I do what I have to to survive.”*

The above quote echoes dominant masculine protector traditions, where it is clear some suicidal men aim to cope via connection, carving out a sense of purpose and self-worth amidst the hopelessness and burdensomeness of suicidal distress [15]. Notwithstanding this, the pandemic context was cited as prohibitive of the effectiveness of men’s usual coping strategies: “*during the pandemic my usual coping measures failed to work*”. Echoing the “Aussie battler” sentiment [56], and mirroring current quantitative findings on the universally protective nature of resilience, other participants conveyed an emphasis on “*hanging in there*” and “*inner strength*”. This suggests daily mental strength and persistence were required in finding any sense of mental stability in the wake of feeling suicidal which was likely amplified by the uncertainty of a COVID-19 milieu.

*“Sheer will. I have no strategies, and all strategies I was taught never work.”*

It was clear in some responses that participants found comfort in the well-worn adage in suicide prevention, that suicide is a permanent solution to a temporary problem. The notion of having a life worth living and *“finding things to look forward to, even if they’re just short term”* reinforced the importance of purposefully cultivating their own future. This sentiment may have arisen considering the restrictive nature of the pandemic context, where participants aimed to hold on until life could return to ‘normal’.

*“My will to live and plans for the future outweighed my desire to end it all.”*

The techniques participants mentioned to help them overcome these periods of heightened distress may align with an array of common therapeutic modalities like mindfulness, positive psychology, cognitive behaviour therapy and dialectical behaviour therapy as participants reminded themselves of the importance of “*trying to be grateful”,* and “*these are just thoughts my brain throws up*” to help them cope.

Alongside the positive mental health coping strategies described above, a range of behavioural factors were also mentioned as protective in this theme. This indicated a sense that participants were focusing on the elements of their life they *could* control amidst a state of distress that was otherwise chaotic in the context of COVID. Strategies here included “*making plans to keep active and eat better*”, reducing substance use (“*12 step program*”) and “*getting enough sleep*”.

Indicative of participants’ efforts to reach out to cope with their suicidal thoughts or behaviours, various forms of help-seeking and engagement with mental health services were also evident in this theme. These were often observed in tandem with many of the above cognitive or behavioural protective strategies. Avenues of help-seeking mentioned could be understood across a spectrum of severity, including safety planning (e.g., “*mitigation plan*”), psychotherapy (e.g., “*engaging with therapy*”; “*DBT skills*”), pharmacotherapy (e.g., “*medications*”), contacting crisis support lines (e.g., “*calling Lifeline*”) and at most severe, hospital admissions to psychiatric units (e.g., “*I was hospitalised. My admission was 52 days*”). Although men’s engagement with mental health services is often framed as wanting a ‘quick fix’ [57], responses here indicated a sense of comfort among participants with the knowledge that their help-seeking might, and likely should be, a long-term management strategy.

*"My mental health professional realised that I was in crisis in June 2021 and arranged for me to be admitted to a secure mental health facility. I spent just under a month there. With ongoing therapy and a readjustment of my medications, I felt the time in care was of enormous benefit."*

Finally, common across responses in this theme was enlisting support from partners, friends, and family, alongside spending time with pets to cope with suicidal distress. Many responses indicated a network of social support across friends and family, with the suicidal man at the centre (e.g., “*my dog and partner with the support of close friends*”), where others simply mentioned “*family, family, family*”; “*my wife*”; “*my kids*”.

Building on the quantitative finding of the protective nature of social support from friends, this also often took the form of activities with friends that could serve as a distraction from distress (e.g., “*game nights with friends*”; “*breaking quarantine to hang out with mates*”). Common across these responses was direct mention of sharing experiences with one’s social circles, where men ostensibly overcame a socialised doctrine of masculine silence in the wake of distress [58]. This prioritisation of protective connections was perhaps facilitated in part by the COVID context, norming and necessitating enlistment of support.

*"Calling a friend. Having a friend that I can talk to is the only thing that's gotten me out of a depressive episode and [the] only thing that's stopped me from killing myself. De-escalating my mental health through being connected."*

It is important to note that across responses in this theme, the various positive coping strategies were interconnected and overlapping, representing a suite of protective factors that drew on elements from each of the areas described above.

#### Sustaining selflessness

One hundred participants provided responses coded under this theme. This theme largely reflected the various ‘in the moment’ factors interrupting men from acting on suicidal thoughts and impulses. This contrasts with the components described above that were framed as protective against suicidal distress more generally. Our sense in interpreting the data, was that responses themed as indicative of *sustaining selflessness* were relevant when men had seemingly exhausted the buffering effects of help-seeking, social support and cognitive resilience. This internal process of considering the ramifications of their actions seemed to run along constantly in the background, connecting these men to their reasons for living and often triggering a cyclical re-energising of attempts to reach in and reach out.

Firstly, responses in this theme indicated the overwhelming mental imagery of a suicide attempt. This was distressing for participants, where it appeared the “*fear of how physically painful the process of dying would be*” was a factor that was protective against suicidal action as there is “*[no] painless way of killing [yourself]*”. Additionally, participants mentioned an awareness and fear of the ramifications of a so-called ‘failed’ suicide attempt “*leaving me a vegetable*”, and not wanting to “*be left more debilitated and disabled than I am now*”. This hints at the often-discussed idea of death by suicide being male terrain indicated in the gender paradox of suicidal behaviour [18]. This fear of a suicide attempt not culminating in death often led to maladaptive coping mechanisms like “*drinking*” and “*hard drug abuse*” as a short-term solution to try to distract from the existential dread of their suicidal thinking. These coping mechanisms could also be indicative of an ambivalence for living and self-care, induced by a state of psychological distress, or an avoidance of confronting the reality of one’s distress (identified as problematic in our quantitative findings).

Furthermore, several participants described suicidal action as “*selfish*”, which connected with arguably the most common protective factor in this theme: the anticipated effects of men’s suicide on those closest to them. Awareness of the negative effects of men’s suicide on those around them was reflected in clear awareness of the “*pain*”, “*trauma*” and “*enormous harm*” this would cause, “*ruin[ing] the lives*” of their family, friends and pets. Mention of pets echoes scholarship on the capacity for animals to ameliorate psychological distress [59]; “*the fact that my dog wouldn’t understand if I was gone after almost 20 years in [their] life*”). Men also reported embodying a carer role in supporting the welfare of their pets (e.g., “*who will look after my animals???*”; “*I do not want my cat to come to any harm after I am gone*”).

These ramifications extended from the emotional repercussions of grief and loss to the financial, with several participants noting the practical matter that they had not yet organised a will. One participant found that ensuring he had a constant reminder of what his actions would mean in “*a photo of my 14-month-old son stuck next to the speedo of my car*” was the form of protection he needed from taking his own life. Some participants had experienced this devastation first-hand, where one had “*attempted in the past and saw the pain it caused, so trying not to put others and family through [that] again*”, and another described “*the effect a suicide of a friend had on me*”. Others reported witnessing and experiencing the despair associated with losing a family member to suicide, and not wanting to re-inflict this on their loved ones:

“*A close family member killed himself 4 years ago. I am afraid of what my actions will do to others.*”

Participants also mentioned avoiding suicidal action due to the effects on first-responders and those involved in a catastrophic suicide attempt. It was clear that no matter how insurmountable participants’ distress seemed, the anticipated despair of their loved ones outweighed and circumvented any suicidal action, echoing selflessness as a health-promoting masculine value [60]. This sentiment also potentially explains one protective component of being in a relationship during the pandemic, as observed in our quantitative findings.

*"The effect it would have on my family and the absolute disastrous effect it would have on the random innocent person that would have been driving a car that I would have driven into at high speed".*

Many participants spoke of occupying a central role as carer, protector and provider in the lives of others, and thus being needed (e.g., “*Still raising my son and cannot let the family down*”; “*my elderly mother depends on me*”; “*the need to protect my Godkids from dealing with a broken home/life from parental separation*”), and to exit this role via suicide would manifest as a detrimental ‘ripple effect’ that would outweigh any sense of distressed burdensomeness with being alive:

*"Ultimately reminding myself that I have children that I want to be there for, and that if I feel I should commit suicide that I have to just stop everything I'm failing at and give up on it, and just be there for family at the very minimum. I gave myself a mental ultimatum that if I decided to kill myself I would just stop everything and get better at all costs."*

Taken together, this theme encompassed the many and varied cognitive processes that inhibit suicidal action. Fear of the act itself, how it would be perceived, and its impact on those in men’s social circles appeared, for many participants, to be the final protective barrier against allowing suicidal thoughts to progress to action.

## Discussion

The aim of the current survey study was to explore associations between key risk and protective factors across the spectrum of suicidal thoughts or behaviours among Australian men in the context of the COVID-19 pandemic. Specifically, the quantitative findings offer unique insights delineating men experiencing no suicidal thoughts or behaviours compared to suicidal ideation and/or planning. Results indicated that men residing in metropolitan areas exhibited lower odds of both suicidal ideation and planning relative to no suicidal thoughts or behaviours. Where married/partnered men were less likely to report suicidal ideation, retaining employment throughout the pandemic and greater social support from friends were protective against suicide planning. Unexpectedly given qualitative reports of the benefits of emotion- and problem-focused coping strategies, higher emotion-focused coping (e.g., venting, acceptance) conferred greater odds of suicidal ideation, and higher problem-focused coping greater odds of suicide planning, where avoidant coping was consistently associated with greater odds of both ideation and planning. Finally, and as echoed in our qualitative findings, greater resilience was protective against both suicidal ideation and planning. No measures significantly delineated odds of suicide planning relative to ideation alone. Qualitative insights however complemented and extended upon these findings, where the first theme highlighted that to mitigate suicidal distress and ideation, men reach in to access internal coping resources, and reach out to enlist social support. The second theme elucidated elements that inhibit suicidal action; potentially in the absence of other protective factors, including the anticipated individual and shared pain of an attempted or completed suicide.

### Protective factors related to suicidal thoughts or behaviours in men

Principally, our findings highlight that even in the wake of experiencing stressors that confer clear risk for suicidal thoughts or behaviours, several protective components can buffer these effects and promote well-being; or at the very least, keep suicidal men from acting on their thoughts. Relative to those who experienced loss of employment in the pandemic, retaining employment appeared protective specifically against suicide planning among men. This aligns with past research focusing on unemployment and suicide risk in men [38, 61]. It also supports the body of work on negative life events, consistent with O’Connor’s IMV model, which highlights the latter as triggers for the emergence of suicidal thoughts and behaviours [14]. The work of Costanza and colleagues [62] during the pandemic found that among patients admitted to psychiatric emergency departments, fear of employment insecurity was more common during ‘lockdown’ periods than post-lockdown. Our results extend this to suggest that COVID-era job loss might have been particularly aversive for some men, given its link with suicide ideation and planning. Prior research examining so-termed ‘economic suicides’ in the wake of the 2008 financial crisis also observed a stronger link between financial stressors and suicidal behaviour in men than women [63]. Importantly, in times of such economic hardship, the work of Economu and colleagues emphasised the protective role of social capital and social connection to others [64]. Specifically, ‘interpersonal trust’, defined as the belief in the inherent goodwill of other people, can be a sole protective factor against suicidal ideation [64] in times of economic crisis. Perhaps, therefore, the degree of social integration afforded to men who retained employment served as a mechanism through which their risk of suicide planning was buffered. Regarding relationships, our results align with past studies suggesting that married/partnered men experienced lesser odds of suicidal ideation alone (but was unrelated to suicide planning; [35]). These findings reinforce the theoretical understanding of the ‘social nature’ of male suicide [16], linking men’s psychosocial context (e.g. employment; relationship status) with distinct categories of suicide risk in men.

Prior COVID-era research found that the most commonly-reported fears among psychiatric inpatients during and after lockdown pertained to containment measures, which gave rise to isolation and loneliness [62]. It follows then that our results build upon research highlighting social support as a key protective factor against suicide in men [31] by exploring associations between various sources of social support and sub-categories of suicide risk. Only perceived social support from friends was associated with lower odds of suicide planning relative to no suicidal thoughts or behaviours. A broad body of literature links greater social support (in general) as protective against suicidal ideation [65], and behaviour [66]; however to our knowledge, ours is the first study to delineate sources of social support in relation to suicide risk in men. Past research has observed crucial differences between individuals who attempt suicide and individuals with no suicidal history, being the number of friends with whom participants had daily interactions [67] and significantly lower level of perceived social support [68].

Qualitative findings help to explain our results regarding the salience of friendship as buffering against suicide risk: it appeared that participants sought social support from friends throughout the pandemic to ameliorate or distract themselves from their distress. Whereas the role of family and significant others became more pronounced in men’s imaginings of the impact of their suicide on loved ones (as also reported by Struszczyk and colleagues [40]). Elements of this narrative contrast past literature highlighting men are more inclined to discuss emotional problems with their partners [69]. Yet perhaps during the pandemic where social connection was largely confined to technology and social-media platforms, in severe contexts where men’s distress progresses to suicide planning, men may be more inclined to seek support from friends. This might be a result of the fear of over-burdening or worrying those closest to them, which might preclude their help-seeking from family or partners (as has been observed in research with fathers; [70]). Moreover, given the unique nature of COVID lockdowns where families were in constant, close confines with one another, this may have shifted the type of support sought and contexts in which men’s help-seeking took place. Regardless, this novel finding requires confirmation in future. Given the known role of social isolation in men’s suicidal thoughts [71], helping men to establish and maintain meaningful social connections when distressed, especially in a pandemic context, could represent an essential suicide prevention measure.

Findings regarding avoidant coping as related to greater odds of both suicidal ideation and planning align with literature describing the maladaptive nature of avoidant coping processes such as substance use [72, 73]. This is especially important as avoidant coping behaviours are often found to be more common in men [74, 75] and align more closely with conformity to traditional masculine norms of self-reliance, risk-taking and dominance [70, 76]. Interestingly, our results suggest higher levels of emotion-focused coping are also associated with *greater* risk of suicidal ideation among men. Perhaps among some men, maintaining a degree of distance from one’s psychological pain (e.g. traditional stoicism) and its source may represent a protective strategy against the escalation of feelings of weakness, hopelessness or lack of control into suicidal thoughts. Notwithstanding this, our quantitative results regarding the association between emotion-focused coping and greater odds of suicidal ideation somewhat contrast qualitative reports of the benefits of such strategies in keeping men safe when experiencing suicidal thoughts and/or behaviour. Perhaps a greater tendency to engage with one’s emotions in the early stages of distress leads to increased rumination and subsequent suicidal ideation in some men. However, as reported in our qualitative findings, when men are already in a suicidal mindset, such emotion regulation strategies may shift towards a protective role against a worsening state of distress. This idea was reflected in qualitative findings where many men described the salutogenic effects of recognising the transient nature of suicidal thoughts and feelings, rather than dwelling on these symptoms; also echoing the protective nature of masculine norms against the exacerbation of depressive symptoms in some contexts (e.g., stoicism; toughness; [77, 78]).

Higher problem-focused coping was also associated with greater odds of suicide planning which similarly contrasts existing findings [40]. However, past work has highlighted the framing of suicide as a masculine act of ‘taking back control’ from a life of distress among some men [46]; and perhaps this finding is therefore explained by the tendency for suicide planning to manifest as a problem-focused coping strategy for certain suicidal men. Our qualitative findings suggested a masculine process of ‘fighting back’ against one’s distress by some men, as protective against suicide. These novel findings regarding coping require replication in future, as there are potentially individual differences at play which determine the extent to which emotion and/or problem-focused coping strategies are helpful or harmful for suicidal men in given contexts and beyond the COVID-19 pandemic.

Qualitative findings highlighted a broad suite of psychological strategies adopted by participants which clearly cultivated resilience against suicidal distress; likely underpinning our finding that greater resilience was protective against both suicidal ideation and planning. Many of these strategies were mentioned alongside other strategies such as help-seeking and the enlistment of social support; this reflects the known synergistic effects of social support and positive life experiences (e.g., success at work) in cultivating resilience and thereby protecting against suicide [79]. The pandemic context may also be relevant here, where the widespread experience of mental ill-health was normalized among the population at large, and many government-funded public health awareness campaigns were run to encourage positive thinking and resilience [80].

### Implications

Many participants described the potential consequences of their death as keeping them safe, ostensibly in the absence of other protections. These findings provide important insights into the components that tether men to life at the most severe end of their suicidal distress. Foremost among these was a desire to hold on to their strength and refrain from passing down trauma onto loved ones via their suicide, mirroring past work highlighting the circuit breaking role of the ‘other’ as a turning point to help-seeking among suicidal men [46]. The protective nature of social connections is pertinent to leverage in future public mental health campaigns delivered during times of crisis. This is especially important given that fears pertaining to social isolation were commonly observed among individuals in severe distress during and post-lockdown [62]. Firstly, for practitioners working with suicidal men, adopting a strengths-based approach to reinforcing men’s self-worth and their instrumental role in the family unit and in society more generally could be an effective means of targeting feelings of hopelessness and entrapment in suicidal men. Additionally, continuing to create non-stigmatising public health campaigns centred on men’s lived experience that realistically communicate the effects of suicide on those left behind, whilst also acknowledging the pain of those in suicidal crisis, may be an effective means of reminding suicidal men of reasons for living, especially if they have exhausted their battery of other potential protective factors [81]. Finally, our findings regarding social support from friends reinforce the value of upskilling supporters as gatekeepers to not only detect suicidal distress in their male friends but also navigate challenging conversations around suicide to best identify when professional intervention is needed. Whilst men often value the distraction from their distress that instrumental social support provides [39]; opportunities to meaningfully engage in frank conversations about the extent of men’s distress should not be avoided by help-givers.

Finally, where prior research has discussed suicide planning as an “active” form of ideation [20], it is rare for studies to effectively delineate between individuals experiencing ideation and those who have progressed to a state of suicide planning. O’Connor and Kirtley [14] also acknowledge that we still know relatively little about the distinction between “passive” and “active” ideation. Therefore, our comparison aiming to delineate those with ideation and planning, from ideation alone, should be repeated in future with a larger sample size and greater depth of planning assessment (as discussed below) to uncover potential distinguishing factors that were not captured in the current study.

### Strengths and limitations

To our knowledge, this study is the first to concurrently examine key suicide risk and protective factors among a sample of men, especially in a pandemic context. We have also extended past literature by delineating the specific risk or protective effects that variables confer on sub-categories of suicide risk (i.e., ideation, planning). However, a larger sample size including those who have experienced suicidal behaviour of different potential lethality is likely needed to more fully determine factors that delineate these risk groups. This will enable a closer test of ‘ideation-to-action’ models of suicide risk, where our focus here was on delineating ideation alone from suicide planning. The cross-sectional design and homogeneity of the sample in certain demographic areas (particularly the lack of transgender men included) represent study limitations. Reliability estimates for the emotion-focused (α = 0.67) and avoidant coping (α = 0.67) subscales were also below ideal cut-offs of 0.7 [82] and results pertaining to these subscales should be interpreted with caution. We do note that other studies involving predominantly young male samples have observed comparably low reliability estimates using the Brief COPE scale (e.g., Poulos et al. also reported an alpha of 0.68 for the avoidant coping scale in a sample of 89% male e-sports athletes; [83]). The low reliability of the emotion-focused and avoidant subscales warrants further study particularly with samples of young men, in case uncontrolled variability in this subsample potentially impacted the reliability of our participants’ responses to these scales.

Additionally, this study involved simple assessments of job loss and relationship breakdown. Especially in a pandemic context, important nuance in the links between these factors and categories of suicidal thoughts or behaviours would be better captured in future through more detailed items. For example, assessing the *quality* of relationship functioning and/or effects of subsequent re-employment (or more generally assessing career interruption) in relation to men’s mental health and suicide risk, as opposed to a dichotomous measurement, is warranted. This also necessitates a larger sample size than was achieved here. Greater detail regarding the assessment of suicide planning is also needed in future research, as binary assessment fails to capture important nuance surrounding the complexities of suicide planning. Specifically, Millner and colleagues [84] discuss the importance of obtaining greater depth in assessment of suicide planning, given the lack of consensus regarding that which actually constitutes a suicide plan, and temporal distinctions between various aspects of suicide planning behaviour. Nevertheless, the items applied in this study were useful in allowing differentiation between suicidal ideation alone relative to ideation with history of planning; such categorisation in relation to protective factors among a sample of men had not been completed to-date.

Additionally, given the nature of the survey advertisements requesting participation from men with self-identified mental health challenges during the pandemic, the extent to which our findings reflect more at-risk samples of men with less insight into their distress is limited. Finally, our application of a qualitative survey item returned a notable breadth of responses. However, greater depth and nuance could be achieved in future by including more items to probe protective factors; or alternatively, conduct in-depth interviews to expand our understanding of this particular topic.

The observation that concurrent ideation with planning was more common than ideation alone in the current sample contrasts with other COVID-era research highlighting ideation alone has been much more common than suicidal intentions or plans [85]. Discordant rates observed in past studies could be attributable to differences in sample populations: where other studies have aimed to canvas rates of suicide sub-categories in the general population, our survey advertisements specifically called for participants who had experienced mental health challenges during the pandemic. It is likely we therefore obtained a more clinically unwell population, with potential influence of selection bias, explaining the proportions of suicidal ideation, planning and attempt observed.

## Conclusions

Findings from this study highlight the many and varied protective factors that keep men safe when experiencing suicidal thoughts or behaviours. Foremost among these was connection to others, be that through an intimate relationship or seeking out social support from friends. Complexity was observed in links between various coping styles and sub-categories of suicide risk; nevertheless, even in the absence of typical protections, suicidal men can and will seek out elements that promote safety and life in the context of overwhelming distress. In all, findings from this study support the necessity of appraising male suicide through an interpersonal lens, where various forms of real or imagined relational support can be integral in saving men’s lives. Such a framing is essential to apply to our investigation of suicide risk and protective factors in the context of global crises like the COVID-19 pandemic, where traditional avenues of support can be restricted.

## Data Availability

The data analysed during the current study are not publicly available due to ethical restraints regarding confidentiality. Data are available from the corresponding author on reasonable request, subject to ethical approval for secondary data analysis.

## References

[1] Naghavi M (2019). Global, regional, and national burden of suicide mortality 1990 to 2016: systematic analysis for the Global Burden of Disease Study 2016. BMJ.

[2] Fazel S, Runeson B (2020). Suicide. N Engl J Med.

[3] Australian Bureau of Statistics (2021) Causes of Death, Australia, 2020 | Australian Bureau of Statistics. https://www.abs.gov.au/statistics/health/causes-death/causes-death-australia/latest-release. Accessed 23 Aug 2022

[4] Richardson C, Robb KA, O’Connor RC (2021). A systematic review of suicidal behaviour in men: A narrative synthesis of risk factors. Soc Sci Med.

[5] Proto E, Quintana-Domeque C (2021). COVID-19 and mental health deterioration by ethnicity and gender in the UK. PLoS ONE.

[6] Wasserman D, Iosue M, Wuestefeld A, Carli V (2020). Adaptation of evidence-based suicide prevention strategies during and after the COVID-19 pandemic. World Psychiatry.

[7] Ellison JM, Semlow AR, Jaeger EC, Griffth DM. COVID-19 and MENtal Health: Addressing Men’s Mental Health Needs in the Digital World. Am J Mens Health. 2021;15(4):15579883211030020. 10.1177/15579883211030021. 10.1177/15579883211030021 PMC8267042 34229530

[8] Pirkis J, Gunnell D, Shin S (2022). Suicide numbers during the first 9–15 months of the COVID-19 pandemic compared with pre-existing trends: An interrupted time series analysis in 33 countries. EClinicalMedicine.

[9] Dubé JP, Smith MM, Sherry SB, Hewitt PL, Stewart SH (2021). Suicide behaviors during the COVID-19 pandemic: A meta-analysis of 54 studies. Psychiatry Res.

[10] Kõlves K, Kõlves KE, de Leo D (2013). Natural disasters and suicidal behaviours: a systematic literature review. J Affect Disord.

[11] Lee SM, Kang WS, Cho AR, Kim T, Park JK (2018). Psychological impact of the 2015 MERS outbreak on hospital workers and quarantined hemodialysis patients. Compr Psychiatry.

[12] Turecki G, Brent DA, Gunnell D, O’Connor RC, Oquendo MA, Pirkis J, Stanley BH (2019). Suicide and suicide risk. Nat Rev Dis Primers 2019.

[13] O’Connor RC (2011). The integrated motivational-volitional model of suicidal behavior. Crisis.

[14] O’Connor RC, Kirtley OJ (2018). The integrated motivational–volitional model of suicidal behaviour. Philoso Trans R So B: Biol Sci.

[15] van Orden KA, Witte TK, Cukrowicz KC, Braithwaite SR, Selby EA, Joiner TE (2010). The interpersonal theory of suicide. Psychol Rev.

[16] Coleman D, Kaplan MS, Casey JT (2011). The social nature of male suicide: A new analytic model. Int J Mens Health.

[17] Burke TA, Ammerman BA, Knorr AC, Alloy LB, McCloskey MS (2017). Measuring acquired capability for suicide within an ideation-to-action framework. Psychol Violence.

[18] Schrijvers DL, Bollen J, Sabbe BGC (2012). The gender paradox in suicidal behavior and its impact on the suicidal process. J Affect Disord.

[19] Bryan CJ, Butner JE, May AM, Rugo KF, Harris JA, Oakey DN, Bryan AO (2020). Nonlinear change processes and the emergence of suicidal behavior: A conceptual model based on the fluid vulnerability theory of suicide. New Ideas Psychol.

[20] Klonsky DE, May AM (2015). The three-step theory (3ST): A new theory of suicide rooted in the “ideation-to-action” framework. Int J Cogn Ther.

[21] Nock MK, Borges G, Bromet EJ, Cha CB, Kessler RC, Lee S (2008). Suicide and suicidal behavior. Epidemiol Rev.

[22] Borges G, Angst J, Nock MK, Ruscio AM, Walters EE, Kessler RC (2006). A risk index for 12-month suicide attempts in the National Comorbidity Survey Replication (NCS-R). Psychol Med.

[23] Nock MK, Borges G, Bromet EJ, Alonso J, Angermeyer M, Beautrais A, Bruffaerts R, Chiu WT, De Girolamo G, Gluzman S, De Graaf R (2008). Cross-national prevalence and risk factors for suicidal ideation, plans and attempts. Br J Psychiatry.

[24] Player MJ, Proudfoot J, Fogarty A, Whittle E, Spurrier M, Shand F, Christensen H, Hadzi-Pavlovic D, Wilhelm K (2015). What Interrupts Suicide Attempts in Men: A Qualitative Study. PLoS ONE.

[25] River J (2018). Diverse and Dynamic Interactions: A Model of Suicidal Men’s Help Seeking as It Relates to Health Services. Am J Mens Health.

[26] Ridge D, Smith H, Fixsen A, Broom A, Oliffe J (2021). How men step back – and recover – from suicide attempts: A relational and gendered account. Sociol Health Illn.

[27] Baca-Garcia E, Perez-Rodriguez MM, Oquendo MA, Keyes KM, Hasin DS, Grant BF, Blanco C (2011). Estimating Risk for Suicide Attempt: Are we Asking the Right Questions? Passive Suicidal Ideation as a Marker for Suicidal Behavior. J Affect Disord.

[28] Witte TK, Merrill KA, Stellrecht NE, Bernert RA, Hollar DL, Schatschneider C, Joiner TE (2008). “Impulsive” youth suicide attempters are not necessarily all that impulsive. J Affect Disord.

[29] Moscardini EH, Pardue-Bourgeois S, Oakey-Frost DN, Powers J, Bryan CJ, Tucker RP (2021). Suicide Cognitions Scale: Psychometric Support in a Community Sample Using Bifactor Modeling and Altered Item Content. Assessment.

[30] Ribeiro JD, Franklin JC, Fox KR, Bentley KH, Kleiman EM, Chang BP, Nock MK (2016). Self-injurious thoughts and behaviors as risk factors for future suicide ideation, attempts, and death: a meta-analysis of longitudinal studies. Psychol Med.

[31] Richardson C, Robb KA, McManus S, O’Connor RC (2022) Psychosocial factors that distinguish between men and women who have suicidal thoughts and attempt suicide: findings from a national probability sample of adults. Psychol Med. 10.1017/S0033291721005195. 10.1017/S0033291721005195 PMC10235670 35012702

[32] Khan AR, Ratele K, Arendse N (2020). Men, Suicide, and Covid-19: Critical Masculinity Analyses and Interventions. Postdigital Sci Educ.

[33] Cunningham R, Milner A, Gibb S, Rijnberg V, Disney G, Kavanagh AM (2021) Gendered experiences of unemployment, suicide and self-harm: a population-level record linkage study. Psychol Med. 10.1017/S0033291721000994. 10.1017/S0033291721000994 33875022

[34] Andreeva E, Magnusson Hanson LL, Westerlund H, Theorell T, Brenner MH (2015). Depressive symptoms as a cause and effect of job loss in men and women: Evidence in the context of organisational downsizing from the Swedish Longitudinal Occupational Survey of Health. BMC Public Health.

[35] Evans R, Scourfield J, Moore G (2014). Gender, Relationship Breakdown, and Suicide Risk: A Review of Research in Western Countries. J Fam Issues.

[36] Pietromonaco PR, Overall NC (2021). Applying relationship science to evaluate how the COVID-19 pandemic may impact couples’ relationships. Am Psychol.

[37] Ogrodniczuk JS, Rice SM, Kealy D, Seidler ZE, Delara M, Oliffe JL (2021). Psychosocial impact of the COVID-19 pandemic: a cross-sectional study of online help-seeking Canadian men. Postgrad Med.

[38] Clapperton A, Newstead S, Frew C, Bugeja L, Pirkis J (2020). Pathways to Suicide among People with a Diagnosed Mental Illness in Victoria, Australia. Crisis.

[39] McKenzie SK, Collings S, Jenkin G, River J (2018). Masculinity, Social Connectedness, and Mental Health: Men’s Diverse Patterns of Practice. Am J Mens Health.

[40] Struszczyk S, Galdas PM, Tiffin PA (2019). Men and suicide prevention: A scoping review. J Ment Health.

[41] Gunnell D, Appleby L, Arensman E (2020). Suicide risk and prevention during the COVID-19 pandemic. Lancet Psychiatry.

[42] Roy A, Sarchiapone M, Carli V (2007). Low resilience in suicide attempters. Arch Suicide Res.

[43] Youssef NA, Green KT, Dedert EA, Hertzberg JS, Calhoun PS, Dennis MF, Beckham JC (2013). Exploration of the influence of childhood trauma, combat exposure, and the resilience construct on depression and suicidal ideation among U.S. Iraq/Afghanistan era military personnel and veterans. Arch Suicide Res.

[44] Pienaar J, Rothmann S, van de Vijver F (2007). Occupational Stress, Personality Traits, Coping Strategies, and Suicide Ideation in the South African Police Service. Criminal Justice and Behaviour.

[45] Ambrus L, Sunnqvist C, Asp M, Westling S, Westrin Å (2020). Coping and suicide risk in high risk psychiatric patients. J Ment Health.

[46] Seidler ZE, Wilson MJ, Oliffe JL, Kealy D, Toogood N, Ogrodniczuk JS, Rice SM (2021). “Eventually, I Admitted, ‘I Cannot Do This Alone’”: Exploring Experiences of Suicidality and Help-Seeking Drivers Among Australian Men. Front Sociol.

[47] Oliffe JL, Ogrodniczuk JS, Bottorff JL, Johnson JL, Hoyak K (2012). “You feel like you can’t live anymore”: suicide from the perspectives of Canadian men who experience depression. Soc Sci Med.

[48] Terhaag S, Quinn B, Swami N, Daraganova G (2020) Findings from Ten to Men: the Australian Longitudinal Study on Male Health, 2013–16. Australian Institute of Family Studies

[49] Carver CS (1997). You want to measure coping but your protocol’s too long: consider the brief COPE. Int J Behav Med.

[50] Meyer B (2001). Coping with Severe Mental Illness: Relations of the Brief COPE with Symptoms, Functioning, and Well-Being. J Psychopathol Behav Assess.

[51] Zimet GD, Powell SS, Farley GK, Werkman S, Berkoff KA (1990). Psychometric characteristics of the Multidimensional Scale of Perceived Social Support. J Pers Assess.

[52] Smith BW, Dalen J, Wiggins K, Tooley E, Christopher P, Bernard J (2008). The brief resilience scale: assessing the ability to bounce back. Int J Behav Med.

[53] Chmitorz A, Wenzel M, Stieglitz RD (2018). Population-based validation of a German version of the Brief Resilience Scale. PLoS ONE.

[54] Clarke V, Braun V, Hayfield N. Thematic Analysis. In: Smith J, editor. Qualitative psychology: A practical guide to research methods. Sage Publications; 2015. p. 222–48.

[55] Braun V, Clarke V, Boulton E, Davey L, McEvoy C (2020). The online survey as a qualitative research tool. Int J Soc Res Methodol.

[56] Whitman K (2013). The “Aussie Battler” and the Hegemony of Centralising Working-Class Masculinity in Australia. Aust Fem Stud.

[57] Seidler ZE, Rice SM, Oliffe JL, Fogarty AS, Dhillon HM (2018). Men In and Out of Treatment for Depression: Strategies for Improved Engagement. Aust Psychol.

[58] Chandler A (2021) Masculinities and suicide: unsettling ‘talk’ as a response to suicide in men. Crit Public Health. 10.1080/0958159620211908959

[59] Blazina C, Boyra G, Shen-Miller D (2011) The Psychology of the Human-Animal Bond. The Psychology of the Human-Animal Bond. 10.1007/978-1-4419-9761-6.

[60] Oliffe JL, Rice S, Kelly MT, Ogrodniczuk JS, Broom A, Robertson S, Black N (2019). A mixed-methods study of the health-related masculine values among young Canadian men. Psychol Men Masc.

[61] Schneider B, Grebner K, Schnabel A, Hampel H, Georgi K, Seidler A (2011). Impact of employment status and work-related factors on risk of completed suicide. A case-control psychological autopsy study. Psychiatry Res.

[62] Costanza A, Macheret L, Folliet A, Amerio A, Aguglia A, Serafini G, Prada P, Bondolfi G, Sarasin F, Ambrosetti J (2021). COVID-19 Related Fears of Patients Admitted to a Psychiatric Emergency Department during and Post-Lockdown in Switzerland: Preliminary Findings to Look Ahead for Tailored Preventive Mental Health Strategies. Medicina.

[63] Costanza A, Amerio A, Aguglia A, Serafini G, Amore M, Macchiarulo E, Branca F, Merli R (2021). From, “The Interpersonal Theory of Suicide” to “The Interpersonal Trust”: an unexpected and effective resource to mitigate economic crisis-related suicide risk in times of Covid-19?. Acta Bio Medica: Atenei Parmensis.

[64] Economou M, Madianos M, Peppou LE, Theleritis C, Patelakis A, Stefanis C (2013). Suicidal ideation and reported suicide attempts in Greece during the economic crisis. World Psychiatry.

[65] Calati R, Ferrari C, Brittner M, Oasi O, Olié E, Carvalho AF, Courtet P (2019). Suicidal thoughts and behaviors and social isolation: A narrative review of the literature. J Affect Disord.

[66] Houle J, Mishara BL, Chagnon F (2008). An empirical test of a mediation model of the impact of the traditional male gender role on suicidal behavior in men. J Affect Disord.

[67] Veiel HOF, Brill G, Hafner H, Welz R (1988). The social supports of suicide attempters: The different roles of family and friends. Am J Community Psychol.

[68] Suresh Kumar P, George B (2013). Life events, social support, coping strategies, and quality of life in attempted suicide: A case-control study. Indian J Psychiatry.

[69] Herron Rv, Ahmadu M, Allan JA, Waddell CM, Roger K (2020). “Talk about it:” changing masculinities and mental health in rural places?. Soc Sci Med.

[70] Livingston JD, Youssef GJ, Francis LM, Greenwood CJ, Olsson CA, Macdonald JA (2022). Hidden in Plain Sight? Men’s Coping Patterns and Psychological Distress Before and During the COVID-19 Pandemic. Front Psychiatry.

[71] Oliffe JL, Broom A, Popa M, Jenkins EK, Rice SM, Ferlatte O, Rossnagel E (2019). Unpacking Social Isolation in Men’s Suicidality. Qual Health Res.

[72] Berke DS, Leone R, Parrott D, Gallagher KE (2020). Drink, Don’t Think: The Role of Masculinity and Thought Suppression in Men’s Alcohol-Related Aggression. Psychol Men Masc.

[73] Rice SM, Fallon BJ, Aucote HM, Möller-Leimkühler AM (2013). Development and preliminary validation of the male depression risk scale: furthering the assessment of depression in men. J Affect Disord.

[74] Berke DS, Reidy D, Zeichner A (2018). Masculinity, emotion regulation, and psychopathology: A critical review and integrated model. Clin Psychol Rev.

[75] Tamres LK, Janicki D, Helgeson VS (2002). Sex Differences in Coping Behavior: A Meta-Analytic Review and an Examination of Relative Coping. Pers Soc Psychol Rev.

[76] Wilson MJ, Seidler ZE, Oliffe JL, Toogood N, Kealy D, Ogrodniczuk JS, Walther A, Rice SM (2022). “Appreciate the Little Things”: A Qualitative Survey of Men’s Coping Strategies and Mental Health Impacts During the COVID-19 Pandemic. Am J Mens Health.

[77] Sileo KM, Kershaw TS (2020). Dimensions of Masculine Norms, Depression, and Mental Health Service Utilization: Results From a Prospective Cohort Study Among Emerging Adult Men in the United States. Am J Mens Health.

[78] Salgado DM, Knowlton AL, Johnson BL (2019). Men’s health-risk and protective behaviors: The effects of masculinity and masculine norms. Psychol Men Masc.

[79] Kleiman EM, Riskind JH, Schaefer KE (2014). Social support and positive events as suicide resiliency factors: Examination of synergistic buffering effects. Arch Suicide Res.

[80] Mental health support | Coronavirus Victoria. In: 2022. https://www.coronavirus.vic.gov.au/mental-health-support. Accessed 23 Aug 2022

[81] Kennedy AJ, Brumby SA, Versace VL, Brumby-Rendell T (2020). The ripple effect: A digital intervention to reduce suicide stigma among farming men. BMC Public Health.

[82] Hair JF, Black WC, Babin BJ, Anderson RE. Multivariate data analysis. 2010. Pearson College Division

[83] Poulus D, Coulter TJ, Trotter MG, Polman R (2020). Stress and coping in esports and the influence of mental toughness. Front Psychol.

[84] Millner AJ, Lee MD, Nock MK (2017). Describing and measuring the pathway to suicide attempts: A preliminary study. Suicide and Life-Threatening Behavior.

[85] Staples L, Nielssen O, Kayrouz R, Cross S, Karin E, Ryan K, Dear B, Titov N (2020). Rapid report 2: Symptoms of anxiety and depression during the first 12 weeks of the Coronavirus (COVID-19) pandemic in Australia. Internet Interv.

